# Efficacy and safety of augmented-reality pathway-specific binocular training in patients with unilateral amblyopia (ARPSBT): study protocol for a multicenter randomized controlled trial

**DOI:** 10.1186/s13063-025-08927-2

**Published:** 2025-07-01

**Authors:** Yulian Zhou, Shuyang Guo, Ling Ling, Yige Gao, Xinyan Duan, Yan Liu, Rui Liu, Hu Liu, Huihang Wang, Jing Lin, Chen Zhao, Peng Zhang, Wen Wen

**Affiliations:** 1 Department of Ophthalmology & Visual Science, Eye & ENT Hospital, Shanghai Medical College, Fudan University, Shanghai, 200031 China; 2 State Key Laboratory of Medical Neurobiology and MOE Frontiers Center for Brain Science, Institutes of Brain Science, Fudan University, Shanghai, 200032 China; 3 Key Laboratory of Myopia and Related Eye Diseases, Ministry of Health, Fudan University, Shanghai, 200031 China; 4 Shanghai Key Laboratory of Visual Impairment and Restoration, Fudan University, Shanghai, 200031 China; 5 State Key Laboratory of Brain and Cognitive Science, Institute of Biophysics, Chinese Academy of Sciences, Beijing, 100101 China; 6 University of Chinese Academy of Sciences, Beijing, 100049 China; 7 Department of Ophthalmology, The First Affiliated Hospital With Nanjing Medical University, Nanjing, 210029 China; 8 Department of Ophthalmology, The First Affiliated Hospital of Fujian Medical University, Fuzhou, 350005 China

**Keywords:** Randomized clinical trial, Amblyopia, Binocular therapy, Augmented-reality, Patching

## Abstract

**Background:**

Amblyopia is a common developmental disorder with reduced visual acuity and impairment in binocular functions. Patching the fellow eye, as the gold standard therapy in amblyopia, sometimes fails to achieve satisfactory outcomes because of poor adherence or delayed intervention. Recently, dichoptic/binocular digital therapy in amblyopia has been developed, but no evidence-based binocular treatments with superiority are available. Based on our previous study findings in neural deficits of unilateral amblyopia, we develop a paradigm of pathway-specific visual training using augmented-reality techniques, in which functions of the parvocellular pathway in the amblyopic eye are selectively pushed under binocular viewing. The aim of this trial is to assess the efficacy and safety of this innovative binocular, home-based treatment for children and adults with unilateral amblyopia. We hypothesize that augmented-reality pathway-specific binocular training will significantly improve visual functions compared with traditional patching.

**Methods:**

This is a superiority, multi-center, randomized, controlled trial involving 114 patients, aged between 5 and 55 years, with a diagnosis of unilateral amblyopia. Participants will be randomized 1:1 to either binocular training or patching, and will receive home-based treatment for 2 hours per day over a 13-week period. Their visual functions will be assessed at baseline, mid-treatment (week 2, week 4, week 9), and the end of treatment (week 13). The primary outcome is the change in best-corrected visual acuity at distance in the amblyopic eye from baseline to 13 weeks after treatment. Secondary outcomes include contrast sensitivity, near and far stereoacuity, interocular suppression, treatment adherence, and the incidence of adverse events at each visit.

**Discussion:**

This study is the first multi-center randomized controlled trial investigating a home-based augmented-reality binocular therapy targeting neural deficits in children and adults with unilateral amblyopia. We expect that the trial will generate findings that can provide an evidence-based basis for the efficacy and safety of this innovative approach to amblyopia treatment.

**Trial registration:**

ClinicalTrials.gov identifier: NCT06498206. Registered on July 12, 2024.

**Supplementary Information:**

The online version contains supplementary material available at 10.1186/s13063-025-08927-2.

## Administrative information

Note: the numbers in curly brackets in this protocol refer to SPIRIT checklist item numbers. The order of the items has been modified to group similar items (see http://www.equator-network.org/reporting-guidelines/spirit-2013-statement-defining-standard-protocol-items-for-clinical-trials/).

**Table:** 

Title {1}	Efficacy and safety of augmented-reality pathway-specific binocular training in patients with unilateral amblyopia (ARPSBT): study protocol for a multicenter randomized controlled trial
Trial registration {2a and 2b}.	ClinicalTrials.gov: NCT06498206. [Registered on July 12, 2024]
Protocol version {3}	March 25, 2025, Version1.2
Funding {4}	National Natural Science Foundation of China (82271117 to WW), and Shanghai Science and Technology Innovation Action Plan (22ZR1410200 to WW).
Author details {5a}	^1^Department of Ophthalmology & Visual Science, Eye & ENT Hospital, Shanghai Medical College, Fudan University, Shanghai, 200031 China ^2^State Key Laboratory of Medical Neurobiology and MOE Frontiers Center for Brain Science, Institutes of Brain Science, Fudan University, Shanghai 200032, China ^3^Key laboratory of Myopia and Related Eye Diseases, Ministry of Health, Fudan University, Shanghai 200031, China ^4^Shanghai Key Laboratory of Visual Impairment and Restoration, Fudan University, Shanghai 200031, China ^5^State Key Laboratory of Brain and Cognitive Science, Institute of Biophysics, Chinese Academy of Sciences, Beijing 100101, China ^6^University of Chinese Academy of Sciences, Beijing 100049, China ^7^Department of Ophthalmology, The First Affiliated Hospital with Nanjing Medical University, Nanjing 210029, China. ^8^Department of Ophthalmology, The First Affiliated Hospital of Fujian Medical University, Fuzhou 350005, China.
Name and contact information for the trial sponsor {5b}	Wen Wen, Eye & ENT Hospital of Fudan University, No.83 Fenyang road, Xuhui district, Shanghai, China 200031 Email: wenweneye@126.com
Role of sponsor {5c}	This is an investigator initiated clinical trial. No sponsor played any part in the study design, collection, analysis, interpretation, report writing, publication and other activities related to the study.

## Introduction

### Background and rationale {6a}

Amblyopia is a visual impairment with reduced vision and binocular dysfunctions due to abnormal binocular interaction and/or visual deprivation during the critical period of visual development, without any apparent organic lesion in the visual system. A meta-analysis showed the prevalence of amblyopia was estimated at 1.44% in the global population, and the highest prevalence was found in participants over 20 years old, which was 3.29% [1]. Amblyopic individuals experience a broad range of visual deficits besides reduced visual acuity, such as reduced contrast sensitivity, fixation instability, abnormal binocularity, spatial uncertainty, and impaired reading abilities [2–6]. Amblyopia is an important public health problem because of its prevalence in children, and the visual impairment as well as psychosocial issues from amblyopia can have a lifelong and profound impact on life quality if it is not treated effectively.

Patching the fellow eye (FE) is the gold standard for the treatment in amblyopic children, requiring patients to regularly wear an eye patch over the non-affected eye. It works by inhibiting the use of the FE, thereby forcing the use of the amblyopic eye (AE) to alleviate the suppression from the FE and improve the visual ability of the AE. However, this traditional method often leads to poor patient adherence due to its impact on daily life and self-esteem, resulting in less-than-ideal therapeutic outcomes and missed optimal treatment opportunities. Meanwhile, traditional patching for amblyopia has a high recurrence rate after a recovery of visual acuity [7, 8] and is mostly aimed at children under the age of about 7 [9]. For older children and adults, traditional patching always achieves limited effect [10], and there is a general belief that they have missed the critical period for visual recovery [9]. However, new therapies have emerged to find the way to address the drawbacks of traditional therapies, such as perceptual learning [11], binocular video games [12], and transcranial magnetic stimulation [13]. These methods also challenge the previous notion that amblyopia cannot be treated after the end of the critical period.

Contrary to the traditional view of "monocular amblyopia", studies in recent decades have revealed that amblyopia is a "binocular disorder", in which the stronger the inhibition of the AE from the FE, the greater the impairment in binocular functions [14]. Based on this new understanding, investigators have discovered binocular/dichoptic therapies with the hypothesis that rebalancing the inhibitory signals between the two eyes can minimize the suppression of the cortical input in the AE and thus promote the brain to learn to "see" through the AE [15]. Unlike previous monocular training, binocular visual training requires the simultaneous use of both eyes but different visual stimuli to each eye, not only to improve monocular vision of the AE but also to enhance the binocular functions, which should theoretically be a priority over the traditional patching [14]. Binocular/dichoptic training paradigms with reduced contrast in the FE's image and/or binocularly complementary images have been reported, aimed at rebalancing binocular inputs and enhancing binocular sensory fusion [15–17]. However, existing binocular training methods have some barriers to success, including unclear neural mechanisms, repeated visual stimuli or limited visual content, occupation of daily study or work time, and very little effect in older children and adults. So far, there is no evidence-based binocular treatment with superiority compared to patching available.

In this trial, we propose an innovative dichoptic training paradigm using the augmented reality (AR) technique [18], and compare the therapeutic outcomes between traditional patching and AR push-pull binocular training with randomized controlled design. This binocular training method has two major innovation points. Firstly, we design the training paradigm based on neural deficits we previously found. Using high-resolution fMRI technique, we found that stimuli presented to the AE shows selectively reduced response in the parvocellular (P) layers of the lateral geniculate nucleus and weaker effective connectivity to the early visual cortex V1 compared to magnocellular (M) layers [19]. Moreover, imbalanced binocular suppression and weakened binocular integration in the superficial layers of V1 using submillimeter 7 T fMRI, coexisting with attenuated and delayed monocular processing in V1 layers receiving thalamic input in human amblyopia [20]. These precise neural deficits help us to develop more targeted and effective treatments for amblyopia. Therefore, we combine the M-P pathways push-pull in the AE to selectively strengthen the P pathway in the AE, with the interocular P-P pathways push-pull to selectively strengthen the AE under binocular viewing condition. Secondly, we use augmented-reality technique and dichoptic head-mounted displays to achieve environment interactivity and free choice of visual content when training. AR technology processes the scenes in the real surrounding world in real time, extending or augmenting the reality by adding images, sounds, videos or other virtual details, with the virtual environment and the real environment coexisting and interactive, which enables participants to interact with the surrounding environment while performing diverse visual tasks, and also makes long-term training possible.

Our preliminary study with a prospective cohort (Zhou Y. et al., unpublished data) found innovative AR push-pull binocular training method showed effectiveness and safety in adults with unilateral amblyopia. The effective proportion was 60% (15/25) after treatment for 3 months, 2 hours per day, with effectiveness defined as improvement of at least 2 logMAR lines in best-corrected visual acuity (BCVA) at distance in the AE. Since the treatment response is believed age dependant in amlyopia, this method may bring benefits to patients with unilateral amblyopia including children and adults, which is a promising alternative in patients with treatment-resistant amblyopia or with poor adherence to patching. Therefore, in this multicenter, randomized controlled clinical trial, we evaluate the efficacy and safety of this AR training treatment compared with traditional patching in children and adults with unilateral amblyopia, and provide evidence for its further clinical application.

### Objectives {7}

The objective of this clinical trial is to evaluate the efficacy and safety of the AR binocular treatment we developed. The primary objective is to compare the total effective proportion, defined as the proportion of patients with improvement of BCVA at distance in the AE no less than 0.2 logMAR, after 13 weeks of AR binocular training with that of traditional patching in patients with unilateral amblyopia. The secondary objectives of this study are (1) to compare the improvement in the visual functions at each follow-up visit between the two groups, including habitual-corrected visual acuity at distance, contrast sensitivity, near and far stereoacuity, and interocular suppression, (2) to compare the treatment adherence and adverse events between the two groups.

### Trial design {8}

This study is a multicenter, superiority, parallel randomized controlled trial using a 1:1 parallel control design. A total of 114 patients with unilateral amblyopia will be enrolled in 4 centers (Shanghai Eye & ENT Hospital of Fudan University, First Affiliated Hospital of Fujian Medical University, Shanghai Tongren Hospital of Shanghai Jiao Tong University School of Medicine, and Jiangsu Provincial People's Hospital) in China. All patients are stratified and randomly assigned to the intervention (AR pathway-specific binocular training) group and the positive control (patching) group according to age. Based on full-time refractive correction, patients receive AR binocular training or patching therapy 2 hours per day, 7 days per week for 13 weeks for a total of 182 hours. The consistency in treatment exposure time between interventions strengthens the validity of direct comparisons in efficacy and safety, controlling for potential confounding effects of dosage variations. Patients will be followed up at 2, 4, 9, and 13 weeks (± 3 days) after enrollment, during which examiners are masked to treatment group assignments, whereas patients and study coordinators who administered interventions were not masked. The primary outcome is the change of BCVA at distance in the AE at 13 weeks measured by the electronic Early Treatment Diabetic Retinopathy Study (ETDRS) chart with Tumbling E target.

## Methods: participants, interventions, and outcomes

### Study setting {9}

Initiated and led by Shanghai Eye & ENT Hospital of Fudan University, this study was launched in August 2024 and planned to enroll 114 patients with unilateral amblyopia from 4 centers (Shanghai Eye & ENT Hospital of Fudan University, First Affiliated Hospital of Fujian Medical University, Shanghai Tongren Hospital of Shanghai Jiao Tong University School of Medicine, Jiangsu Provincial People's Hospital) in China over 12 months (anticipated recruiment duration). Each patient and/or the guardian was required to sign an informed consent prior to enrollment. Each center will apply to the Ethics Committee for ethical review.

### Eligibility criteria {10}

This study planned to enroll patients in the outpatient of each participating center. Patients were screened and enrolled under quiet conditions in the outpatient, and those who were younger than 18 years old should be accompanied by a guardian.

#### Inclusion criteria

Patients must meet all of the following criteria to be enrolled in this trial:

1. Age: 5 to 55 years (inclusive);
2. BCVA: Interocular difference of 0.2 logMAR or more, with BCVA in the AE < 1.0 logMAR and BCVA in the better eye within age-normal limits;
3. Risk factors of amblyopia, such as anisometropia, strabismus, deprivation;
4. Patients with deprivation amblyopia must have undergone surgical interventions to address the underlying structural causes of deprivation (e.g., cataract extraction with intraocular lens implantation, ptosis correction surgery, etc.);
5. Optical refractive correction in full time, if needed, for no less than 3 months;
6. Orthotropia (orthotropia with refractive correction or after strabismus surgery) or intermittent exotropia with good fusional control;
7. No ongoing or other planned amblyopia treatment (except spectacles).

#### Exclusion criteria

Patients could not be included in this trial if they met any of the following criteria:

1. Ongoing structural pathology in the visual system (e.g., ptosis, media opacity, nystagmus, paracentral fixation, optic nerve diseases like glaucoma, retinal diseases, cortical visual impairment, etc.);
2. History of ocular surgery that affects vision (except surgery to remove the causative factors of amblyopia, such as strabismus surgery and congenital cataract surgery);
3. Significant systemic disorders (e.g., epilepsy, cognitive impairment);
4. Implantable electronic device;
5. Ongoing or planned use of medications that may affect vision (except dilating eye drops e.g. atropine used for optometry examinations);
6. Those who have diplopia and can not achieve binocular fusion when using the AR binocular training devices;
7. Pregnant or breastfeeding women, or women planning to become pregnant.

### Who will take informed consent? {26a}

The target population was patients with unilateral amblyopia, who come to the centers and meet the eligibility criteria. For patients meeting the eligibility criteria, the investigator must provide them with the details of this clinical trial prior to enrollment, including the contents and purpose of the trial, intended efficacy, possible adverse events, and corresponding countermeasures. The patients can only be enrolled when they have fully understood this trial and signed the informed consent form. For child participants aged 5 to < 8, parents or guardians in accordance with the law need to provide written informed consent for participation. For child participants aged 8 to < 18, they should also give oral informed consent besides written informed consent signed by parents or guardians. For adult participants, they need to sign the written informed consent form by themselves. The informed consent form is developed in accordance with the Declaration of Helsinki, which will be in duplicate and jointly signed by the investigator and the participant and/or his or her guardian, each copy for each party. Participants can withdraw from the study any time they want with no effect on their subsequent care. Informed consent has already been evaluated by the Ethics Committee of Eye & ENT Hospital of Fudan University (Ethical code: [2024]No.2024114).

### Additional consent provisions for collection and use of participant data and biological specimens {26b}

N/a.

Explanation: all patient information, biological samples, and imaging data required for the exploratory endpoints of this study were included in the formal informed consent and were fully informed by the investigators at the time of enrollment.

## Interventions

### Explanation for the choice of comparators {6b}

A total of 114 patients were randomly divided into the following two groups according to age (≥ 18 years old, < 18 years old): (1) AR pathway-specific binocular training group and (2) patching group.

### Intervention description {11a}

#### AR pathway-specific binocular training system

**Fig. 1:**
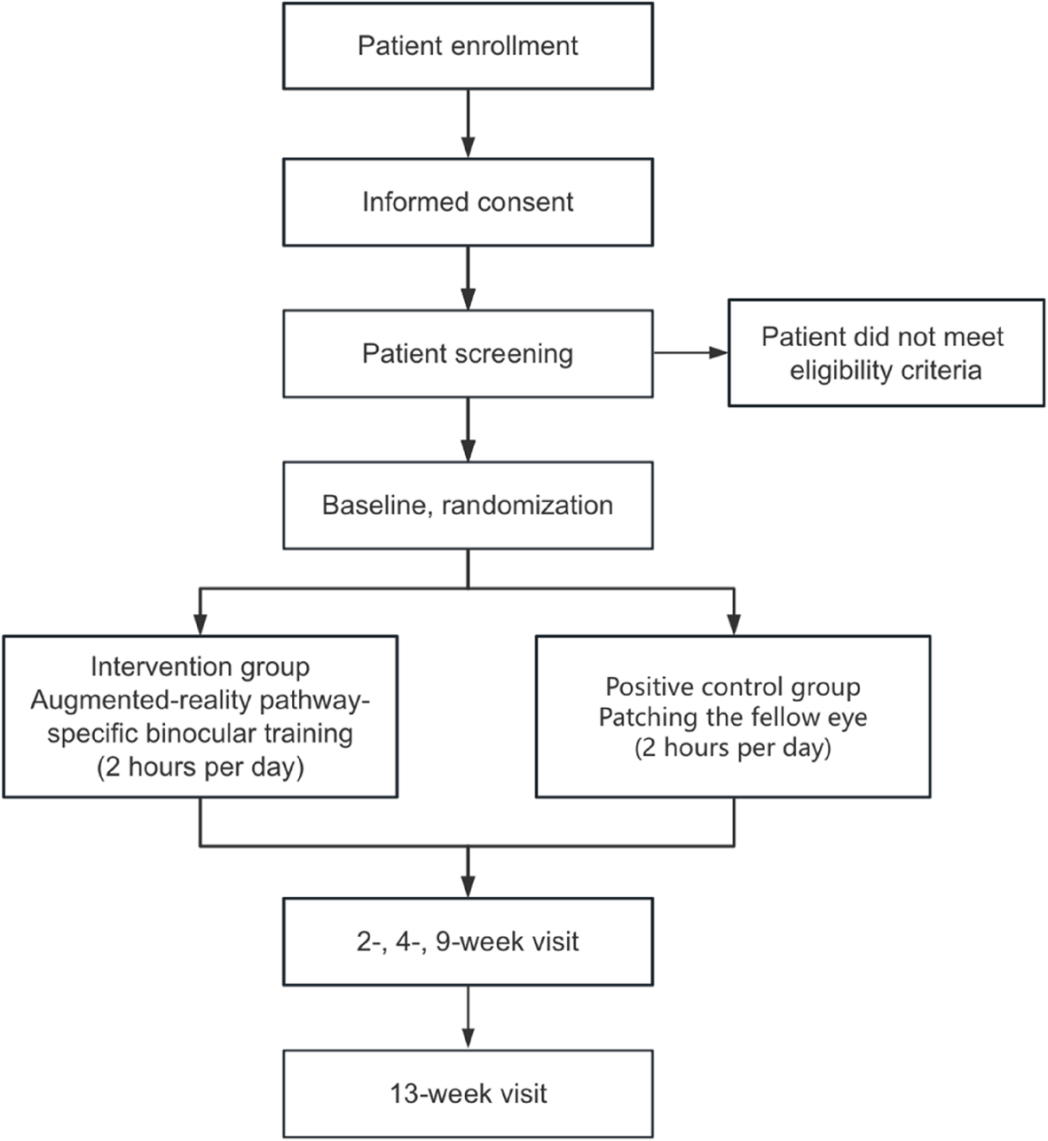
Schematic of AR pathway-specific binocular training. **a** Dichoptic images processing in the amblyopic eye and fellow eye. **b** In this demo, the participant was playing a video game while wearing AR glasses. Right panels show the processed images presented to the amblyopic eye and fellow eye

We develop AR binocular training for amblyopia treatment, including original hardware and software. Environmental images are captured through the high-speed and high-resolution camera, which are processed by the graphic process unit of a laptop and displayed on the head-mounted dichoptic displays in real time, allowing the patients to interact with the surrounding environment during the visual training. Using the AR technique, images were processed differently and presented dichoptically to the AE and the FE simultaneously, the same in content but different in contrast, spatial frequency, and temporal frequency (Fig. 1). To selectively enhance the P pathway, which is notably impaired in amblyopia, the low spatial frequency components of visual input to the AE are phase-scrambled into fast-flickering noise with reduced contrast, while the high SF details remain intact with full contrast, to achieve push-pull in monocular P-M pathways in the AE (Video S1). Meanwhile, the high-frequency components of visual input to the FE are processed into slow-flickering noise with a low signal-to-noise ratio (Video S2), to achieve push-pull in interocular P-P pathways between the AE and the FE. More details about the design of the AR pathway-specific binocular training paradigm have been reported in our study published recently [18].

The AR binocular training system has passed the test conducted by Centre Testing International Group Co., Ltd., a leading third-party testing and certification organization in China, accredited with China Metrology Accreditation and China National Accreditation Service for Conformity Assessment certifications. Therefore, this investigational medical device has been certified to meet the prerequisites for conducting a clinical trial. This Class II innovative medical device has been accepted into the Expedited Review Program for Innovative Medical Devices by Zhejiang Medical Device Evaluation Center. According to the national regulations, ethical requirements, and the characteristics of the investigational product, the efficacy and safety of the hardware and software involved in this treatment will be verified.

#### Intervention group

In the intervention group, participants will be required to have AR pathway-specific binocular training at home for 2 hours per day, 7 days per week for 13 weeks, wearing head-mounted dichoptic displays. The backend of the training system will record participants' daily training duration for adherence analysis.

#### Positive control group

In the control group, participants will be required to patch the fellow eye at home for 2 hours per day, 7 days per week for 13 weeks. The daily actual patching duration should be recorded by the participants or their guardians in the diary cards provided.

Participants in both groups are instructed to engage in the same visual tasks during treatment sessions, such as watching video, reading e-books/paper books and comics. For the AR training group, these contents are projected onto the AR glasses' displays, whereas patients in the patching group view them directly. All centers use fabric patches (non-adhesive, wrapping the spectacle lens) to ensure consistent light-blocking efficacy.

### Criteria for discontinuing or modifying allocated interventions {11b}

#### Withdrawal criteria and procedure

1. Withdrawal at the investigator's discretion:under circumstances where the investigator decides to withdraw the case from the trial since the patient enrolled is not suitable for further study during the trial, mainly including:
  i. Serious adverse event or intolerable adverse event during the trial;
  ii. Comorbidities, complications, or significant physiological changes during the trial;
2. Self-withdrawal of the patient from the trial, including:
  i. Unwillingness to continue the clinical trial;
  ii. Loss to follow-up.

The investigators must make a detailed evaluation of the patient withdrawn from the trial due to unexpected adverse events, take active treatment measures, document the entire process in detail, and make frequent follow-ups until the condition is under control. Patients withdrawn from the trial due to personal reasons unrelated to the trial can receive clinical routine treatment, and the routine treatment of patients after the withdrawal will not be affected by this study.This study does not involve intervention modifications. All data of participants randomized are preserved in the EDC system to ensure analytic completeness under the intention-to-treat (ITT) principle, while protocol deviations like non-adherence are documented and analyzed separately.

### Strategies to improve adherence to interventions {11c}

Investigators can verify the treatment adherence of patients by checking their daily training records through the backend of the training system in the AR binocular training group or their daily record cards in the patching group. Training or patching for 2 hours per day will be considered as having completed the treatment for that day, with less than 2 hours considered not reaching an effective dose. The investigator should inform the patient to try to avoid an interruption in training; if there is an interruption, the backend of the system stops the timing, but the time period of training in the same day will be accumulated. During the training process, the system will record the training duration and completion status. If patients do not complete the effective dose for two consecutive days, the researcher will remind them by phone. The abnormal training data detected in the backend for two consecutive days will be verified by phone and recorded truthfully.

The adherence will be calculated based on the actual number of days the treatment of 2 hours completed at each follow-up visit. In order to ensure the adherence to interventions as well as correct and effective training, the following methods are adopted to guide and monitor patients' daily treatment: (1) assistance from doctors and trainers: a guide video is sent to the patients to help them to master the entire operation procedure and to inform important cautions. If patients have any questions about the treatment process, they can contact the doctors or trainers to solve problems through online communication; (2) family assistance: for patients aged under 18, the doctor and trainer will train their family to understand the operation at the baseline visit; (3) incentive measures: for each enrolled patient, we promise to give one year of AR binocular training device use right after the end of the study to encourage them to actively complete the trial.

### Relevant concomitant care permitted or prohibited during the trial {11d}

Ongoing or planned amblyopia treatment (except spectacles) was prohibited. Ongoing or planned use of medications that may affect vision (except dilating eye drops, e.g., atropine used for optometry examinations) was prohibited.

### Provisions for post-trial care {30}

In case of any probable adverse event, the study treatment will cease and appropriate medical protection will be provided by the research team for compensating probable harm.

### Outcomes {12}

#### Primary outcomes

The total effective proportion of amblyopia treatment at week 13. The effective amblyopia treatment is defined as at least 0.2 logMAR improvement of the BCVA at distance in the AE from baseline after treatment, measured with cycloplegic refraction using electronic ETDRS chart. The effective proportion will be calculated for each group.

Effective proportion (%) = Total number of patients meeting the criteria for effective amblyopia treatment/Total number of patients receiving amblyopia treatment in the analysis set × 100.

#### Secondary outcomes

Secondary outcomes included the mean change from baseline to week 13 in the habitual-corrected visual acuity at distance in the AE, the contrast sensitivity in each eye, the stereoacuity at near and distance, the interocular suppression measured by worth-4 dot at near and distance, and the interocular suppression measured by Bagolini striated lens at near and distance, the treatment adherence and safety reports. Additional outcomes included all the measures at week 2, 4, and 9 for the analysis of within-person changes over time.

### Participant timeline {13}

**Fig. 2:**
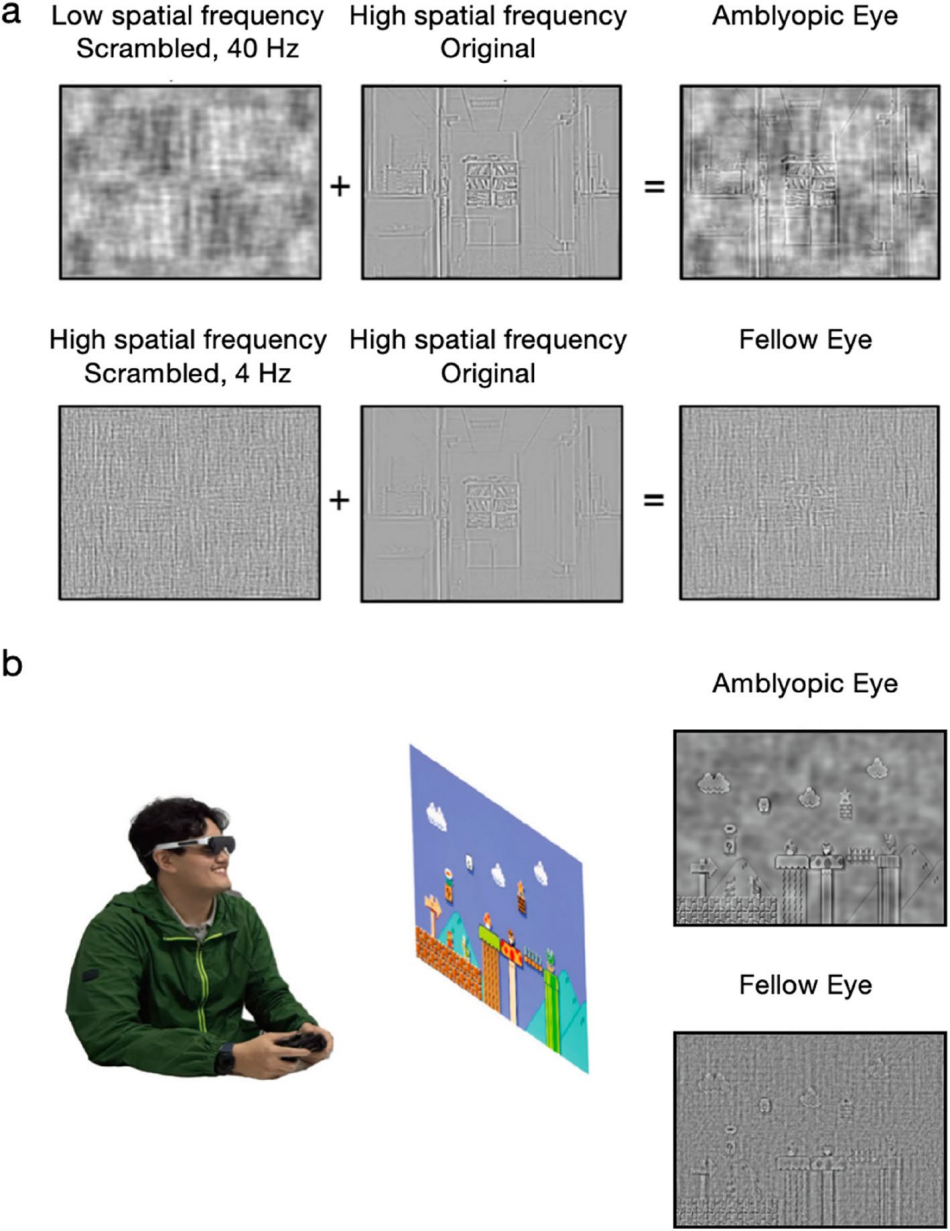
Schematic of the study procedure

The schematic of the study procedure is displayed in Fig. 2.

#### Screening and eligibility assessment

Potential participants are identified from patients coming to the ophthalmology department of the participant centers. Based on the patients' informed consent, medical history and ophthalmic examinations will be evaluated by the investigators for the eligibility screening. Medical history includes a history of ocular diseases, amblyopia treatment and other significant diseases (such as tumor, hypertension, diabetes, heart disease, and epilepsy), a history of ocular surgery, and a history of other major surgeries within the past year. In addition, patients who can not achieve binocular fusion (e.g., uncorrected strabismus) when using AR binocular training devices will be excluded. Ophthalmic examinations are performed for amblyopia diagnosis and patient screening, including the anterior segment examination, fundus photography and optical coherence tomography, prism and alternate cover test, ocular motility, refractive error (cycloplegic refraction in aged ≤ 14), best-corrected visual acuity at near (for presbyopes) and distance, interocular suppression, and stereoacuity. Participants were required to have stable visual acuity before enrollment, defined as wearing the appropriate and same glasses for at least 12 weeks until five or fewer letters change in 2 consecutive visual acuity measurements in the AE (at least 4 weeks apart).

#### Baseline and follow-up assessments

The evaluation schedule of the study is displayed in Table 1. All the measurements are finished by the masked examiners at each visit.

**Table 1:** Summary of trial design

Item\schedule	Study duration					
	Enrollment screening and baseline measurement	Randomization	Treatment period			
	Visit 1 Day − 7 ~ Day 0	Visit 2 Day 0	Visit 3 2 weeks ± 3 days	Visit 4 4 weeks ± 3 days	Visit 5 9 weeks ± 3 days	Visit 6 13 weeks ± 3 days
Allocation		X				
Anterior segment	X					X
Fundus check	X					X
Prism and alternate cover test	X					X
Ocular motility	X					X
Worth 4-dot	X		X	X	X	X
Bagolini striated lens	X		X	X	X	X
Randot Stereotest	X		X	X	X	X
Visual acuity	X		X	X	X	X
Contrast sensitivity	X		X	X	X	X
Axial length	X		X	X	X	X
Refraction	X		X	X	X	X
Adherence	X		X	X	X	X
Adverse events	X		X	X	X	X

Visual acuity in each eye will be measured with the ETDRS chart with Tumbling E target at 4 m (uncorrected visual acuity and habitual-corrected visual acuity) and the electronic ETDRS with Tumbling E target at 2.5 m (BCVA). Contrast sensitivity in each eye will be tested at 2.5 m with the CSV-1000® instrument (VectorVision, Greenville, OH, USA) in a darkroom, for 4 frequencies in cycles per degree (cpd), including 3, 6, 12, and 18 cpd. Interocular suppression will be evaluated by the Worth-4 dot test and Bagolini striated lens test at 33 cm and 6 m. Stereoacuity will be tested by the Randot stereotest (Stereo Optical Co., Inc., Chicago, IL) at 40 cm and 3 m. The anterior segment examination, fundus check (fundus photography and optical coherence tomography), binocular alignment (prism and alternate cover test) and ocular motility evaluation (nine cardinal positions of gaze for function evaluation of extraocular muscles) will be performed only at the baseline and final visit. Other evaluations including refractive error (cycloplegic refraction in aged ≤ 14), axial length, adherence, and adverse events will be performed at each visit.

$$\mathrm{Adherence}\;(\%)=(\mathrm{Completed}\;\mathrm{treatment}\;\mathrm{days}/\mathrm{Scheduled}\;\mathrm{treatment}\;\mathrm{days})\times100$$

The patient adherence is graded as "excellent", "good", "fair", and "poor" when ranging between 76-100%, 51-75%, 26-50%, and 25% or below, respectively. Moreover, at the final visit after completing the treatment, both investigators and patients in the AR training group will evaluate the satisfaction with the software's functions and ease of use.

Safety reports assessment of the types (adverse event, serious adverse event, device deficiency), incidence rate (%), and frequency (number of events) of adverse events and device-related adverse events, serious adverse events and device-related adverse events, and device deficiency occurring during the clinical trial.

### Sample size {14}

According to the requirements of the Good Clinical Practice for Medical Devices ([2022] No. 28) and the Guidance for the Design of Medical Device Clinical Trials, sample size estimation will be performed referring to the clinical study results of comparable medical devices of the investigational medical device and clinical expert opinions.

In this study, the sample size will be calculated based on the primary endpoint, namely the total effective proportion at 13 weeks, using the superiority design. Considering the treatment effects are strongly age-dependent, the participants are divided into 2 groups based on ages: Subgroup I (aged 5 to under 18 years) and Subgroup II (aged 18 to under 55 years).

Subgroup I: Based on clinical expert opinions and preliminary study results (unpublished data), the anticipated effective proportion at 13 weeks of the intervention group will be 75%, while that of the control group, based on previous literatures [10, 21, 22] and clinical expert opinions, will be 40%. With a superiority margin of 0 and a 1:1 ratio of intervention group to control group, the sample size required for each group is 28, as calculated using the PASS 2022 software, at the 5% significance level (two-sided) with 80% power. Considering the 15% dropout proportion during the study, 33 patients will be included in the intervention group and the control group, respectively, in patients aged 5 to under 18 years.

Subgroup II: Based on expert opinions and preliminary study results (unpublished data), the anticipated effective proportion at 13 weeks of the intervention group will be 60%, while that of the control group, based on previous literature [23, 24] and clinical expert opinions, will be 20%. With a superiority margin of 0 and 1:1 ratio of intervention group to control group, the sample size required for each group is 20 as calculated using the PASS 2022 software, at the 5% significance level (two-sided) with 80% power. Considering the 15% dropout proportion during the study, 24 patients will be included in the intervention group and the control group, respectively, in patients aged 18 to under 55 years.

In summary, this clinical study is expected to enroll a total of 114 participants, with 57 patients (33 patients in Subgroup I and 24 patients in Subgroup II) in each group. The formula for calculation of sample size is shown as follows:

$$n_{T} = n_{C} = \frac{{\left( {Z_{1 - \alpha /2} + Z_{1 - \beta } } \right)^{2} \left[ {P_{C} \left( {1 - P_{C} } \right) + P_{T} \left( {1 - P_{T} } \right)} \right]}}{{\left( {\left| D \right| - \Delta } \right)^{2} }}$$

*P*_*T*_ and *P*_*C*_ are the expected event proportions of the intervention group and control group, respectively;$\left| D \right|$ is the absolute value of the difference between the expected proportions of the two $\left| {P_{T} - P_{C} } \right|$; Δ is the superiority margin.

### Recruitment {15}

Patients were recruited from the outpatient of the participating centers, where there were considerable ophthalmic patients and professional conditions for amblyopia evaluation and diagnosis.

## Assignment of interventions: allocation

### Sequence generation {16a}

With age (5– < 18 years old, 18– < 55 years old) as a stratified factor, patients will be assigned sequentially into “intervention group” and “positive control group,” according to the randomization sequence generated by the statistician using the PROC PLAN procedure in SAS at a 1:1 ratio.

### Concealment mechanism {16b}

The treatment allocation will be done using a web-based randomization system Clinflash Cloud (https://cloud.clinflash.com) and undertaken by the investigator. Physicians conducting examinations and data analysts were masked to group assignment. Patient masking is not possible in this study; therefore, no code breaking procedures were required.

### Implementation {16c}

With age (≥ 18 years old, < 18 years old) as the stratified indicator, all eligible patients who had signed the written informed consent after a detailed explanation about the trial were randomly divided into two groups respectively named “AR training group” and “positive control group,” at a 1:1 ratio according to the randomization sequence generated by the statistician.

## Assignment of interventions: blinding

### Who will be blinded {17a}

Physicians who conduct follow-up examinations and data analysts cannot know the grouping of patients, physicians receive professional training about visual function evaluation involved in this trial, and staff outside the research team input the data into the Electronic Data Capture (EDC) system. The statisticians cannot know the grouping during data analysis.

### Procedure for unblinding if needed {17b}

Participants cannot be blinded due to significant differences in interventions in this trial. No procedure for unblinding will be needed as all data analyses are performed after the trial is closed.

## Data collection and management

### Plans for assessment and collection of outcomes {18a}

Demographic data, present and past history on amblyopia diagnosis and treatment, present and past history on medication usage and surgery, systemic and mental diseases will be recorded at the assessment of eligibility. Multiple follow-up assessments included visual acuity, contrast sensitivity, binocular depression, and stereoacuity. Follow-up schedules will be the same for both groups. Baseline data and the 13-week visit included follow-up assessments mentioned above and other assessments including anterior segment examination, axial length, fundus photography, and optical coherence tomography, prism and alternate cover test, and ocular motility. The research schedule is shown in Table 1.

### Plans to promote participant retention and complete follow-up {18b}

A comprehensive informed consent process will be conducted to ensure participants fully understand the trial objectives, procedures, and follow-up requirements. The importance of completing the whole follow-up will be emphasized. We will provide participants with a 13-week follow-up schedule and encourage them to visit the clinic on the assigned date. Two days before the follow-up date, the Clinical Research Coordinator (CRC) will proactively remind the patients via phone. Visit dates are negotiated with participants within ± 3 days of the protocol-specified target window to balance scientific rigor and logistical feasibility. A final follow-up assessment, identical in content to the originally scheduled evaluation for that time point, will be conducted prior to therapy cessation if feasible.

### Data management {19}

The Internet-based data management system, the EDC system, was used for data entry, coding, security, and storage. This study will be participant to confidentiality agreements with staff, the data manager (DM), who manages EDC data and shall not modify or delete existing data without authorization. Unauthorized personnel cannot access EDC systems. The DM will design case report forms (CRFs) aligned with the study protocol and implement an EDC system for centralized data management. A data management plan made by the DM will govern all data handling procedures. A Data Validation Plan (DVP) will be developed pre-database activation, specifying system-generated automated queries and manual verification scopes. Manual verification will be carried out in schedule according to the DVP. A pre-lock review will be conducted to validate database completeness and accuracy prior to database lock.

Paper-based CRFs will be used for initial data collection. On the day of each follow-up visit, the PI will review and sign off on paper records to confirm validity before same-day EDC entry, and the CRC will input the data into the EDC system. Original paper documents will be archived securely. The Clinical Research Associate (CRA) will check the source data to ensure consistency with the contents on the electronic CRFs. All EDC data will be exported, encrypted, and archived on read-only optical discs for permanent retention post trial, complying with the Good Clinical Practice (GCP) and regulatory requirements.

### Confidentiality {27}

Patients and researchers are required to sign a confidentiality agreement to ensure that the data were restricted to the use for this project, to ensure the privacy and security of patient data, and to promise that the collected patient data will not be used for any other purpose without written permission.

### Plans for collection, laboratory evaluation and storage of biological specimens for genetic or molecular analysis in this trial/future use {33}

Not applicable.

## Statistical methods

### Statistical methods for primary and secondary outcomes {20a}

The normal quantitative variables will be described as the mean and standard deviation (SD), and other quantitative variables will be described using the median and interquartile range. Categorical variables are described as the number of cases and the percentage of each category. Independent-sample T test or Wilcoxon rank-sum test will be used for the comparison between groups of quantitative variables based on the data distribution characteristics, and chi-square test or exact probability test will be used for categorical variables.

Statistical analysis will be performed using the SAS 9.4 (or above). Unless specified, all statistical tests will be two-sided at the 5% significance level, and the difference is considered statistically significant when the p value is ≤ 0.05. The analysis will be performed on an intention-to-treat and also per-protocol approaches. No interim analyses are planned.

#### Analysis of primary endpoint

For the evaluation of the primary efficacy endpoint, the effective propotions (Wilson 95% CIs) and the between-group difference in effective proportions (Newcombe 95% CIs) at 13 weeks within Subgroups I and II are calculated. If the lower limit of the 95% CIs for the between-group difference in effective proportions within Subgroups I and II are > 0, then superiority is established. Primary analysis will calculate clinical response rates (Wilson 95% CIs) and rate differences (Newcombe 95% CIs) for treatment vs. control arms in Groups I/II at Week 13. Cochran-Mantel–Haenszel (CMH) analysis stratified by center will provide MH-adjusted proportion differences with 95% CIs for between-group comparisons. For the primary efficacy endpoint, results based on both the full analysis set (FAS) and per protocol set (PPS) are provided.

#### Analysis of secondary endpoints

The secondary efficacy endpoints are analyzed based on the FAS and PPS. The statistical methods for secondary efficacy endpoints follow the general principles of statistical analysis described above.

#### Analysis of safety endpoints

Safety endpoints are analyzed based on the safety set (SS). The occurrence type, frequency, severity, correlation with the investigational device of adverse events, and device deficiencies during the trial will be listed, and between-group comparisons are conducted using the chi-square test or Fisher's exact test.

#### Analysis datasets

Three statistical datasets will be used in this clinical trial. The efficacy analysis will be carried out on the basis of FAS and PPS; all baseline demographic data analysis will be performed on FAS; and the safety evaluation will be conducted on the SS.

1. FAS: refers to all participants who have signed the informed consent and are randomly enrolled to participate in the treatment according to the ITT principle;
2. PPS: a subset of the FAS, generally referring to participants who complete the evaluation on the primary endpoint based on the FAS without major protocol violations;
3. SS: all enrolled cases who have already used the device and undergone safety evaluation are included in the SS, regardless of whether they have completed the entire follow-up plan or not.

### Interim analyses {21b}

There are no interim analyses planned.

### Methods for additional analyses (e.g., subgroup analyses) {20b}

The subgroups (aged 5 to < 18; ≥ 18) will be analyzed in both primary and secondary outcomes to test the consistency. The statistical methods follow the general principles of statistical analysis described above.

### Methods in analysis to handle protocol non-adherence and any statistical methods to handle missing data {20c}

In accordance with the principle of ITT analysis, we will analyze the results of the primary outcome by participant randomization, regardless of patients' adherence to the intervention. The worst-case imputation method will be used to handle missing data for primary efficacy data.

### Plans to give access to the full protocol, participant-level data, and statistical code {31c}

The full protocol, de-identified datasets, and statistical code may be made available from the corresponding author upon reasonable request and appropriate resources after the final publication of results.

## Oversight and monitoring

### Composition of the coordinating center and trial steering committee {5d}

Daily support for the trial is provided by:

1. Principal investigators: supervise the trial and are responsible for the medical responsibility of the patients, identify potential recruits, and take informed consent;
2. Sub-investigators: are responsible for the medical responsibility of the patients, identify potential recruits, register the trial registration;
3. Contract Research Organization (CRO): develop the monitoring plan and monitor the conduct of the trial, assign qualified CRA to conduct regular on-site monitoring visits to the participant centers, so as to ensure that all the contents of the study protocol are strictly observed, and check the source data to ensure consistency with the contents on the electronic CRF;
4. CRA: supervise the adherence of the investigators with the protocol, the GCP and relevant laws and regulations in the conduct of clinical trial, the signing of informed consent, screening, rights and interests and safety guarantee of the subjects, the management and use of the investigational medical device, the management of adverse events and device deficiencies, the reporting of safety information, and the recording of clinical trial data and completion of eCRF;
5. DM: design case report forms (CRFs) aligned with the study protocol and implement an EDC system for centralized data management. A DVP will be developed by the DM pre-database activation, specifying system-generated automated queries and manual verification scopes.
6. CRC: assist the PIs in performing daily management tasks of clinical trials, including the recruitment, informed consent, data collection, eCRF completion, and document management, organize data capture, ensure that trial operations comply with GCP and protocol requirements, coordinate participant follow-up visits, and communicate with CRA;
7. Physicians: conduct follow-up examinations.

The study team meets biweekly. There is no trial steering committee or stakeholder and public involvement group.

### Composition of the data monitoring committee, its role and reporting structure {21a}

An external independent body (CRO) will monitor the quality of the study, which have professional knowledge of clinical medicine and health statistics, and be familiar with relevant laws and regulations of clinical trials. And it is independent of the sponsor and has no competing interests. The committee monitors the eligibility, safety, integrity and effectiveness of data through the EDC system. The problem found in recruitment quality, data quality, protocol adherence, and adverse events will be discussed and solved in the team meets.

### Adverse event reporting and harms {22}

The safety of the participants will be monitored for the duration of the 13-week intervention and follow-up period. Potential adverse events in this study include diplopia, headaches, nausea, eye strain, the examination data for new or worsening heterotropia (increase of ≥ 10 prism diopters from baseline), and worsening of VA in either eye (decrease of ≥ 2 lines from baseline). All adverse events (AEs), defined as any unfavorable or unintended medical occurrences during the trial (irrespective of causality or expectedness), will be non-systematically assessed by investigators through structured interviews at follow-up visits. Identified AEs will be promptly documented in outpatient medical records at the follow-up visit and subsequently entered into the EDC system within 24 h of detection. To standardize terminology, AEs will be classified using the Medical Dictionary for Regulatory Activities (MedDRA) and analyzed descriptively, including frequency, severity, and causality assessment. All AEs, whether expected or unexpected, device-related or unrelated, will be fully disclosed in trial publications. When a serious adverse event (SAE) occurs, the investigator should provide adequate treatment immediately and follow it up until recovery or remission is confirmed. SAE data will be reported at regular meetings monthly.

### Frequency and plans for auditing trial conduct {23}

The research team convenes on a monthly basis to review the progress of the trial and address any potential issues that may arise. Furthermore, an independent monitor will verify the presence and integrity of the documentation every two months, which includes informed consent, eligibility criteria, as well as source data collection.

### Plans for communicating important protocol amendments to relevant parties (e.g. trial participants, ethical committees) {25}

Any important amendments to the protocol must receive a favorable opinion from the Eye & ENT Hospital of Fudan University before implementation. All modifications to the protocol need to be communicated to all participating investigators in the trial. Any modification that alters participant coverage or affects the benefits, risks, and limitations of the study will require a new information sheet and consent form. All protocol amendments will undergo review and approval.

### Dissemination plans {31a}

The findings of this study will be fully disclosed in international peer-reviewed journals. Reports includes both positive and negative results.

## Discussion

This is the first randomized controlled trial using the AR binocular push-pull training in amblyopic patients for a wide range of age. Patching is the golden standard for the treatment of amblyopia in children under 7 years old [9]. However, poor adherence, limited improvement of visual functions, and regression after recovery of visual acuity have been observed in the management of amblyopia using conventional patching [7, 8]. For older children and adults, the efficacy of the patching is greatly discounted after the critical period due to the gradual maturation of the visual system [9, 10]. Effective therapy is still lacking in older children and adults who have missed or failed to correction during the critical period. Recently, dichoptic/binocular digital therapy has been developed. However, binocular methods previously reported [15–17] have some shortcomings, including unclarified neural mechanism, a fixed location for the screen, repeated visual tasks, and a cost of time in daily life, preventing long-term adherence in pediatric patients who are under pressure from schoolwork and adults who suffer more social and familial responsibilities and resulting in unsatisfactory outcomes. So far, there is no widely accepted binocular treatment with superiority available for children and adults with amblyopia [25]. Here, we designed an innovative binocular therapy using AR training, based on neural deficits in amblyopia, to achieve better outcomes.

In previous studies of amblyopia pathology, a reduction in sensitivity to fine visual information (of high spatial frequency) has been observed in amblyopic patients [14]. This finding has been attributed by the majority of previous researchers to a functional reduction in the AE and to severe inhibition from the FE. However, recent subcortical studies based on high-field fMRI have revealed that while there is an overall reduction in the AE, this reduction is more pronounced in signals originating from the P pathway, whereas no significant difference from the M pathway has been observed [19]. Advancing to previous research, this finding identifies the specific focal pathway of visual impairment in amblyopia, thereby providing crucial theoretical support for the development of the training system that can effectively enhance the response of the P pathway in amblyopia patients.

In light of the recent discovery of P pathway impairment in amblyopia and the limitations of relying on a fixed place and visual tasks for correction, we have developed a novel visual training system to specifically enhance the P pathway and incorporates cutting-edge AR technology. This method permits not only a more targeted training of the P pathway in patients with amblyopia, but also allows the patient to correct amblyopia while performing activities of daily life through the AR system which enables patients to interact with the real-world environment during training. The visual content is no longer limited to fixed visual stimuli such as gratings, but is integrated into the work, study or entertainment in daily life, which may significantly improve patient adherence. Therefore, this approach has the potential to markedly enhance the efficacy and adherence of training.

## Trial status

The current protocol is version 1.2, March 25, 2025. The trial was registered on July 7, 2024, and the recruitment began on August 31, 2024. The study aims to include 114 participants, and 43 patients have been enrolled. Patient recruitment is estimated to be completed by August 31, 2025.

## Supplementary Information

Supplementary Material 1. SPIRIT Checklist.

Supplementary Material 2: Video S1.

Supplementary Material 3: Video S2.

## Data Availability

The datasets analyzed during the current study are available from the corresponding author on reasonable request.

## Abbreviations

FE: Fellow eye
AE: Amblyopic eye
AR: Augmented reality
P: Parvocellular
M: Magnocellular
BCVA: Best-corrected visual acuity
ETDRS: Early Treatment Diabetic Retinopathy Study
EDC: Electronic Data Capture
CRC: Clinical Research Coordinator
DM: Data manager
CRF: Case report form
DVP: Data Validation Plan
FAS: Full analysis set
PPS: Per protocol set
SS: Safety set
ITT: Intention-to-treat
CRO: Contract Research Organization
CRA: Clinical Research Associate
GCP: Good Clinical Practice
SAE: Serious adverse event
